# The complete mitochondrial genome of *Clepsis pallidana* (Lepidoptera: Tortricidae) with phylogenetic implications

**DOI:** 10.1080/23802359.2022.2041122

**Published:** 2022-02-16

**Authors:** Xueling Song, Ruiqin Dong, Wenxu Yang, Haili Yu

**Affiliations:** a Shaanxi Key Laboratory for Animal Conservation, College of Life Sciences, Northwest University, Xi’an, PR China; b Key Laboratory of Resource Biology and Biotechnology in Western China (Northwest University), Ministry of Education, Xi’an, PR China

**Keywords:** Mitogenome, Tortricidae, Tortricinae, *Clepsis pallidana*

## Abstract

The leaf roller, *Clepsis pallidana* (Fabricius, 1776) (Lepidoptera: Tortricidae), is an important pest of many crops in China. The circular genome is 15,679 bp in length, consisting of 13 protein-coding genes, 22 transfer *RNA* genes, two ribosomal *RNA* genes, and a non-coding AT-rich region. The base composition of the whole mitogenome is 41.2% A, 40.1% T, 11.1% C, and 7.7% G, which shows a strong AT bias. Phylogenetic analysis based on 13 PCGs showed that *C. pallidana* was closely related to *Epiphyas postvittana* and clustered within Archipini clade, which was consistent with the traditional classification.

The leaf roller, *Clepsis pallidana* (Fabricius, 1776), a member of Tortricinae (Lepidoptera: Tortricidae), is a polyphagous moth distributed in the Palearctic Region. Its larvae feed on young leaves and apical buds (Farahbakhsh 1961; Liu and Li 2002; Sun et al. 2021). A total of 34 host plants belonging to 13 families have been recorded for this species from East Asia, Iran, and Europe (Farahbakhsh 1961; Liu and Li 2002; Sun et al. 2021; Yasuda 1975; Byun et al. 1998; Sohn 2002; Jinbo and Nakatani 2003; Trematerra and Baldizzone 2004). Many of them are important crops, such as *Gossypium hirsutum* L., *Fragaria ananassa* Duch., *Glycine max* (L.), and *Malus pumila* Mill. To acquire a more comprehensive understanding of *Clepsis pallidana*, we present the complete mitogenome of *C. pallidana* as the first mitogenome of the genus *Clepsis*.

The specimen of this study was neither vertebrates nor regulated invertebrates. And no ethical issues are involved. The study has been granted an exemption from requiring ethical approval by the Committee on the Ethics of Animal Experiments of Northwest University, Xian, China. The specimen used in this study was collected from Taibai County (33°14′N, 107°18′E), Shaanxi Province, China in July 2018 and deposited at Herbarium of Northwest University, China under the voucher number 2018016 (www.nwu.edu.cn; contact Yu Haili, yuhaili@nwu.edu.cn). Whole genome sequencing was conducted on Illumina HiSeq 2000 platform with 2 × 150 bp pair-end (GENEWIZ Biotech Co. Ltd., Suzhou, China). After removal of the low-quality reads and adapter region, the trimmed reads were assembled by Novoplasty-NOVOplasty version 3.0 software package (Dierckxsens et al. 2017) and annotated by Geneious R8 (Biomatters Ltd, Auckland, New Zealand) under the default parameter settings. PCGs and tRNAs were compared with the sequences in NCBI database to ensure their exact regions. Further, the tRNAscan-SE was used to verify the veracity of tRNA genes (Lowe and Eddy 1997).

The complete mitogenome of *Clepsis pallidana* is 15,679 bp in length, consisting of 13 protein-coding genes, 22 transfer *RNA* genes, two ribosomal *RNA* genes, and a non-coding AT-rich region. The overall base composition of mitogenome is A (41.2%), C (11.1%), G (7.7%), and T (40.1%), with a total of A + T content of 81.3%. The AT-skew and GC-skew of this genome were 0.0156 and −0.1821, respectively. Gene overlaps were found in 5 locations and their total length was 21 bp. The longest overlap was 8 bp in length and resided between *trnW* and *trnC*. The length of 22 tRNAs ranged from 64 bp (*trnI*) to 71 bp (*trnK*), A + T content ranged from 71.8% (*trnK*) to 92.6% (*trnE*). All of these tRNAs can be folded into a canonical cloverleaf secondary structure except for *trnS1*, and its dihydrouridine (DHU) arm consisted of a simple loop. The *16S rRNA* (1,400 bp) was located between *trnL1* and *trnV*, and *12S rRNA* (782 bp) resided between *trnV* and AT-rich region, and their A + T contents were 85.1 and 85.9%, respectively. The non-coding region was located between *12S rRNA* and *trnM* genes with length of 659 bp, and the A + T content was 92.3%. Among the 13 PCGs, *nad1*, *nad4*, *nad4L*, and *nad5* were encoded on the light strand (L-strand), while the remaining nine genes were encoded on the heavy strand (H-strand). The A + T content of these 13 PCGs ranged from 72.4% (*cox1*) to 91.4% (*atp8*). The *cox1* initiated with CGA as the start codon, *nad6* started with ATA, *atp8* and *nad3* started with ATC, *nad2* and *nad5* started with ATT, and the remaining seven PCGs (*cox2*, *atp6*, *cox3*, *nad4*, *nad4l*, *cytb*, and *nad1*) started with ATG. Nine PCGs terminated with conventional stop codons TAA, while *nad4*, *nad5*, *cox1*, and *cox2* used incomplete codon (T—) as termination codon.

We constructed the phylogenetic relationships based on 13 PCGs from 38 species in Tortricidae, with two outgroup species (Figure 1). Thirteen PCGs were aligned with the L-INS-i algorithm as implemented in the MAFFT (Katoh and Standley 2013). Bayesian inference (BI) was performed with MrBayes version 3.2.6 (Ronquist et al. 2012) with the partitioned models determined by PartitionFinder version 2 (Lanfear et al. 2017). The maximum-likehood (ML) phylogenetic analysis was performed with 1,000 bootstrap replicates based on the GTR + F+R5 model in the IQ-TREE (Guindon et al. 2010; Nguyen et al. 2015). As a result, *Clepsis pallidana* was closely related to *Epiphyas postvittana* and clustered within Archipini clade. They clustered to the branch of *Choristoneura + Archips*, and then grouped with two species of *Adoxophyes*. Monophyly for the clade of Archipini is strongly supported in BI tree, the core of these four genera is unambiguously assigned to the tribe based on a brush of hairs from the venter of the uncus in the male genitalia and a curved, spine-shaped signum with a capitulum in the female genitalia.

**Figure 1.**
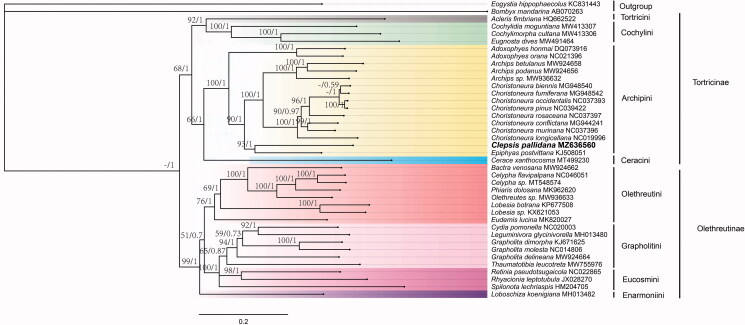
Phylogenetic trees inferred from maximum likelihood and Bayesian inference methods based on the 13 PCGs. Numbers separated by a slash (/) on a node represent the bootstrap support and posterior probability, respectively. The specie with mitogenome sequenced in this study is marked in bold. All GenBank accession numbers of each species were listed in the phylogenetic tree. *Eogystia hippophaecolus* (KC831443) and *Bombyx mandarina* (AB070263) were used as outgroup.

## Author contribution

The authors confirm contribution to the article as follows: study conception and design: Haili Yu, Xueling Song, Ruiqin Dong; data collection: Wenxu Yang; analysis and interpretation of results: Xueling Song, Ruiqin Dong, Wenxu Yang, Haili Yu; draft manuscript preparation: Xueling Song, Haili Yu; revising it critically for intellectual content: Xueling Song. All authors reviewed the results and approved the final version of the manuscript. All authors agree to be accountable for all aspects of the work.

## Data Availability

The genomic sequence data supporting the study's findings are publicly available in NCBI's GenBank https://www.ncbi.nlm.nih.gov/under join number. MZ636560.The associated BioProject, BioSample, and SRA numbers are PRJNA785360, SAMN24563661, and SRR17088790, respectively.
